# The Case of a Janitorial Supervisor With Occupational Asthma Complicated by the Mixed Colonization of the Respiratory Tract by Candida albicans and Alternaria spp.

**DOI:** 10.7759/cureus.14702

**Published:** 2021-04-26

**Authors:** Po Hsuan Huang, Dennis L Caruana, Jonathan Li, Anthony Szema

**Affiliations:** 1 School of Medicine, California University of Science and Medicine, San Bernardino, USA; 2 School of Medicine, Yale University, New Haven, USA; 3 Internal Medicine/Pediatrics, Christiana Care Health System, Wilmington, USA; 4 Allergy and Immunology, Donald and Barbara Zucker School of Medicine at Hofstra/Northwell, Hempstead, USA

**Keywords:** occupational asthma, mixed fungal colonization, dupilumab, reslizumab, biological modifier therapy

## Abstract

Patients on immunosuppressant agents, including oral corticosteroids, are susceptible to fungal colonization despite being otherwise immunologically intact. This case report highlights a state-of-the-art biological modifier therapy for the complex care of a patient with refractory occupational asthma, allergic rhinitis, and mixed fungal colonization.

A dyspneic 38-year-old male janitor with steroid-dependent occupational asthma refractory to omalizumab therapy was colonized with *Candida* and *Alternaria* following a promotion to a managerial position in a moldy office. He was treated with itraconazole and systemic steroids. Pulmonary workup revealed moderate obstructive ventilatory defect, peripheral airway hyperresponsiveness, and eosinophilic airway inﬂammation. Three additional biological modiﬁers (reslizumab, benralizumab, and dupilumab) were administered to this patient. His asthma was ultimately controlled with reslizumab and dupilumab. Fractional exhaled nitric oxide (FeNO) normalized after dupilumab monotherapy, enabling the patient to taper off chronic prednisone therapy.

Various occupational exposures are crucial epidemiologic factors related to infection and go hand-in-glove with long-term prednisone treatment towards increasing susceptibility to fungal colonization. Steroid-sparing anti-interleukin-5 (IL-5) agents and dupilumab should be considered as alternative treatment options for patients, such as ours, with eosinophilic, prednisone-dependent asthma refractory to omalizumab therapy.

## Introduction

Occupational exposure to cleaning and sterilizing agents is a known cause of occupational asthma. The occupational exposome is vastly greater among professional cleaners compared to that in the domestic setting. Additionally, cleaning workers in the healthcare setting are at an even higher risk of developing respiratory symptoms because the cleaning agents they use are required to have stronger antimicrobial and disinfecting properties. A history of atopy, presence of high serum immunoglobulin E (IgE), poorly-controlled asthma, and occupational exposures are all characteristics of sensitizer-induced asthma, which is an occupational asthma subtype wherein aerosolized irritants promote eosinophilic inflammation [1-4]. Cleaning agents have specifically been shown to induce asthma through both sensitizer (immunogenic) and irritant effects. For example, benzalkonium chloride, a common antimicrobial cleaning agent, has been shown to induce IgE and eosinophilic inflammation [5-6]. Janitors are also often exposed to aerosolized dust mite and mold antigens, such as *Dermatophagoides pteronyssinus* and *farinae*, as well as *Penicillium* and *Aspergillus* spp., which have also been implicated in occupational asthma [7-9].

Patients on immunosuppressant agents, which include oral corticosteroids for occupational asthma, are susceptible to fungal colonization despite being otherwise immunologically intact [10]. Patients with eosinophilic, prednisone-dependent asthma have been found to have increased local eosinophil-producing processes, which respond well to anti-interleukin-5 (IL-5) therapy [11]. In 2017, reslizumab was recommended as an add-on therapy for severe eosinophilic asthma by the Global Initiative for Asthma; it has been shown to reduce pulmonary eosinophilia in animal models without inducing immunosuppression [12]. If reslizumab is not available, other anti-IL-5 monoclonal antibodies and other biological modifiers, such as dupilumab (FDA approval granted in 2018), could also be considered as an alternative treatment option for this patient population. This case report highlights a state-of-the-art biological modifier therapy for the complex care of a patient with refractory occupational asthma, allergic rhinitis, and mixed fungal colonization.

## Case presentation

A 38-year-old male, a former smoker with poorly controlled asthma despite being treated with fluticasone/salmeterol or budesonide/formoterol with spacers, and year-long oral prednisone therapy, presented to a community-based outpatient clinic with dyspnea. His past medical history consisted of chronic sinusitis, nasal polyposis status post-sinus surgery, and multiple hospitalizations for phlegmatic dyspnea and pneumonia. Percutaneous skin prick testing was significant for *Dermatophagoides farinae* and *pteronyssinus*, and his serum IgE had been found to be 2,000 IU/ml during a previous hospitalization.

He had worked for many years as a janitor in professional healthcare settings, with occupational exposure to fumes from industrial cleaning agents. Recently, the patient had been promoted to a supervisory position and he had moved into a moldy office where he had subsequently developed a chronic cough, dyspnea, and wheezing.

**Figure 1:**
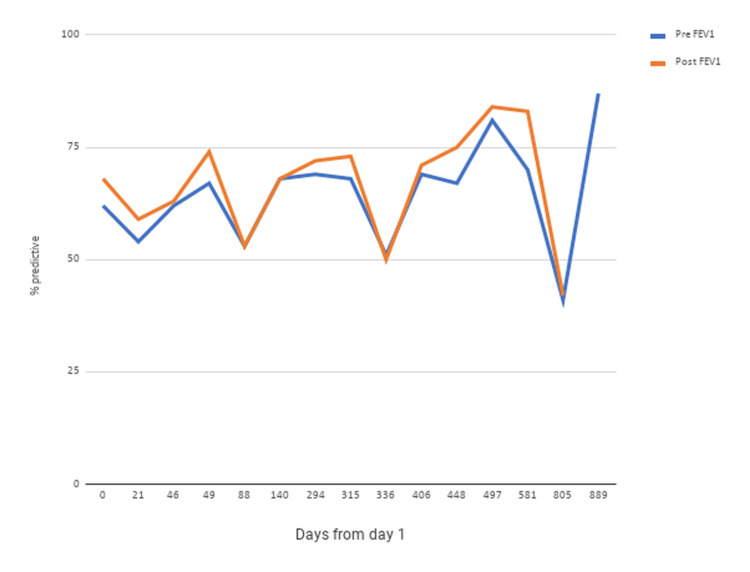
FEV1 pre- and post-bronchodilator studies Spirometry pre- and post-bronchodilator FEV1 values showed an obstructive ventilatory defect. The FEV1 after nebulizer treatment with albuterol improved to nearly 90% of the predicted value after the patient was on reslizumab for three months. From November 18, 2016, to December 8, 2017, the patient was on omalizumab. The patient was on reslizumab from December 8, 2017, to March 9, 2018, on benralizumab from March 9, 2018, to December 7, 2018, and on dupilumab from February 5, 2018, to April 5, 2019 FEV1: forced expiratory volume in one second

**Figure 2:**
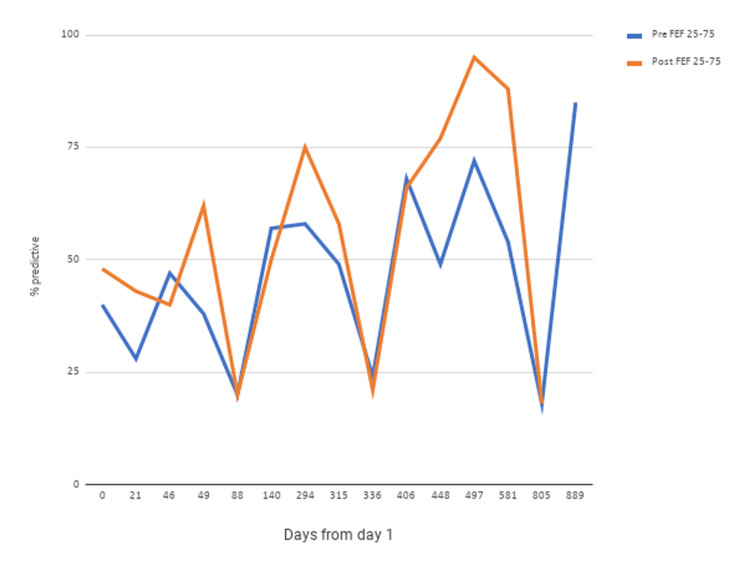
FEF25-75 pre- and post-bronchodilator studies Spirometry pre- and post-bronchodilator FEF25-75 values showed improvement of small airway dysfunction FEF25-75: forced expiratory flow at 25-75%

**Figure 3:**
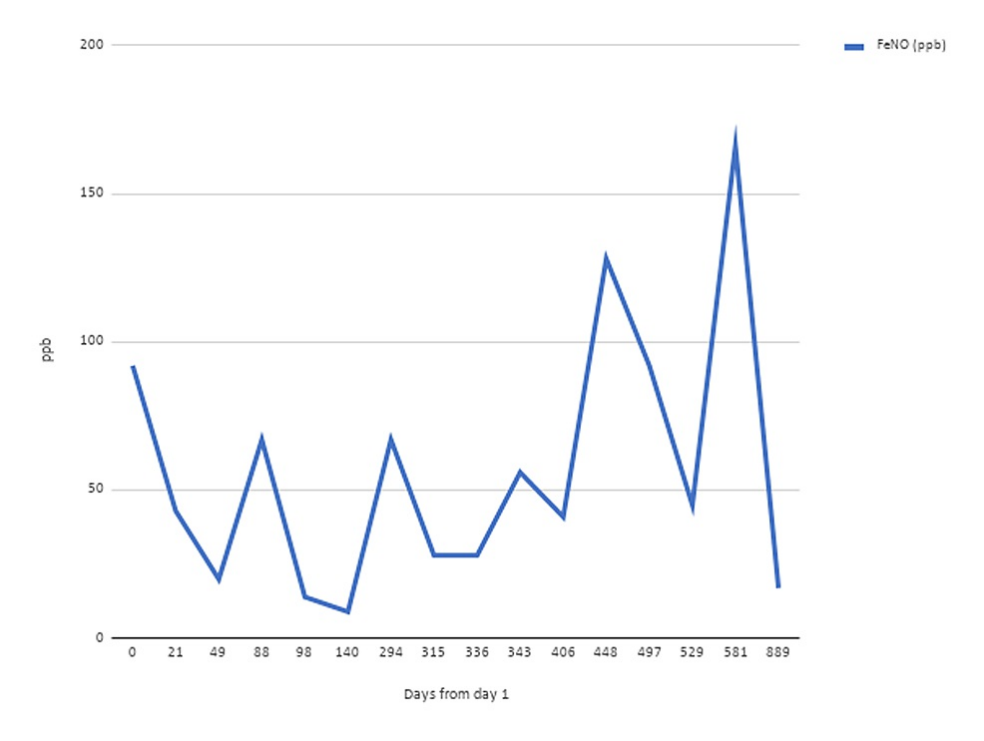
Time-course of FeNO concentration FeNO values were high (>50 ppb) on several occasions despite treatment with omalizumab and reslizumab. FeNO values normalized following dupilumab therapy. Nitric oxide concentration correlates with airway inflammation and reflects eosinophil infiltration into the airway and release of NO, supporting eosinophilic airway inflammations FeNO: fractional exhaled nitric oxide

At his initial visit, his vital signs were stable. His body mass index was 34 kg/m^2^. Upon ambulation, his peripheral capillary oxygen saturation was 95% on room air. Physical exam was significant for diffuse wheeze in all lung fields. Spirometry revealed a moderate obstructive ventilatory defect (Figures 1, 2). Impulse oscillometry revealed an X5, a measure of peripheral airway elastance [13-14] of -1858% predicted (based on height and body mass index), consistent with peripheral airway hyperresponsiveness. Impulse oscillometry also showed an R5-R20%R5 of 32.91% (reference range: ≤20% in adults). R5-R20%R5 is the ratio of peripheral airway resistance (R5-R20) to total airway resistance (R5) wherein R5 and R20 represent the resistance of the respiratory system at 5 and 20 Hz, respectively. Serial fractional exhaled nitric oxide (FeNO) was repeatedly found to be >50 ppb (Figure 3), suggesting eosinophilic airway inflammation. Serum IgE was elevated at 143 IU/ml (reference range: 0-100 IU/ml) without peripheral eosinophilia (2.4%; reference range: 0-3%).

Omalizumab was initiated at 225 mg every two weeks to manage his poorly controlled, steroid-dependent asthma with elevated serum IgE. Over the next year, his response to omalizumab kept fluctuating, requiring steroids at times to control exacerbations. Forced expiratory volume in one second (FEV1) and forced expiratory flow at 25-75% (FEF25-75) still showed only partial or minimal reversal of airway obstruction (Figures 1, 2). He remained chronically dependent on steroids since his symptoms would flare up while tapering. Omalizumab failed to improve the frequency of his asthma exacerbations.

**Figure 4:**
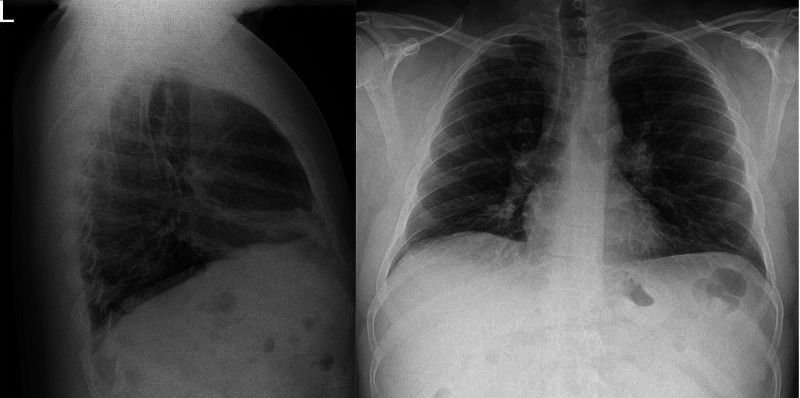
Radiographic demonstration of pulmonary infiltrates Left: lateral chest X-ray that shows that the posterior triangle has infiltrates at the level of the lingula. Right: posterior-anterior view chest X-ray did not demonstrate infiltrates

One year after initiating treatment at our office, the patient presented with green nasal discharge bilaterally and worsening dyspnea. He was afebrile, and the physical exam revealed constant anterior and posterior rhonchi with wheezing throughout all lung fields. A chest X-ray revealed infiltrates in the right lower lobe (Figure 4). Sputum culture grew *Candida albicans* and *Alternaria *spp., which were verified by microscopy with lactophenol cotton blue. Itraconazole and prednisone were initiated as antifungal therapy. The peripheral blood eosinophil percentage increased from 2.4 to 9.6% during this time (reference range: 0-3%), equating to an absolute eosinophil count of 806 cells/μl [4]. Despite improvement in respiratory symptoms, antifungal therapy was discontinued after one month due to itraconazole-induced personality changes. At this time, repeat sputum culture was found to be negative for fungal growth. He had no known history of immunodeficiency (Table 1). To reduce exposure to mold, the patient switched his office to one without visible mold and installed a high-efficiency particulate air filter to improve air quality and reduce aero-allergens.

**Table 1:** Immunoglobulin panel The patient had a normal total IgG level, minimal isolated IgG subclass 2 deficiency, low IgM, and elevated CD4 and CD8 ratio

Test name	Result	Reference range
Immunoglobulin G (IgG), serum	954 mg/dL	694-1,618 mg/dL
Immunoglobulin G, subclass 1	610 mg/dL	382-929 mg/dL
Immunoglobulin G, subclass 2	234 mg/dL	241-700 mg/dL
Immunoglobulin M (IgM)	36 mg/dL	48-271 mg/dL
Helper (CD4)/suppressor (CD8) ratio	5.34	0.86-5.00

Due to his asthma that was refractory to omalizumab, and persistent signs of eosinophilic inflammation (elevated FeNO and peripheral eosinophilia), three different biological modifiers were tried. Reslizumab was initiated at 30 mg every four weeks after omalizumab was discontinued. Pre- and post-bronchodilator values for FEF25-75 and FEV1 improved with three months on reslizumab (Figures 1, 2). Benralizumab, also administered at 30 mg every four weeks after reslizumab, was discontinued for the following reasons: the inconvenience of receiving injections at a local community hospital; worsening of FEF25-75 and FEV1 (Figures 1, 2). Dupilumab was given at 300 mg every two weeks following the failure of benralizumab, and consequently, FEF25-75 and FEV1 returned to normal (Figures 1, 2).

## Discussion

Our patient’s persistently elevated FeNO levels reflected eosinophilic airway inflammation, an indicator of occupational asthma [15]. His airway obstruction in the central and peripheral airways was refractory to steroid and omalizumab therapy. Omalizumab has been well-documented to be efficacious in eosinophilic asthma and non-allergic eosinophilic disease through direct anti-IgE mechanisms and indirect effects on the production of pro-eosinophilic cytokines [16]. Since the patient’s asthma was refractory to omalizumab therapy, it is likely that continued exposure at work had caused his eosinophilic inflammation to persist through non-IgE mechanisms. Due to the persistent need for steroids to control symptoms, even with omalizumab therapy, our patient was colonized by *Candida albicans* and *Alternaria*.

We suspect that mixed fungal colonization arose secondary to immunosuppression from the steroids. Similar cases have been reported in the past with chronic steroid use inducing secondary immunosuppression, leading to invasive fungal pneumonia [17-18]. It must be noted that despite the presence of pulmonary infiltrates at the level of the lingula, the patient did not meet the criteria for invasive fungal infection as outlined by the European Organization for Research and Treatment of Cancer/Invasive Fungal Infections Cooperative Group and the National Institute of Allergy and Infectious Diseases Mycoses Study Group (EORTC/MSG) consensus group [19]. Finally, the patient was not found to have any form of primary immunosuppression.

## Conclusions

Refractory asthma may warrant a pursuit beyond Occam’s razor for more than one diagnosis. Occupational asthma is induced by myriad substances, which trigger airway inflammation through both immunogenic and irritant mechanisms. Atopy, elevated FeNO, and eosinophilia are the emerging hallmarks of this disease. This case demonstrates the critical need to consider the specific exposures at work environments, such as mold. Mold may cause occupational asthma and predispose individuals to colonization by fungal organisms. Having excluded other causes of immunosuppression, we hypothesize that the ingestion of prednisone over a two-year period predisposed our patient to colonization by *Candida albicans* and *Alternaria *spp. Biological modifiers, such as reslizumab and dupilumab, constitute state-of-the-art, steroid-sparing therapies that were successfully utilized in managing our patient’s occupational asthma with eosinophilic inflammation.
